# Feasibility validation of automatic diagnosis of mitral valve prolapse from multi-view echocardiographic sequences based on deep neural network

**DOI:** 10.1093/ehjimp/qyae086

**Published:** 2024-10-28

**Authors:** Zijian Wu, Zhenyi Ge, Zhengdan Ge, Yumeng Xing, Weipeng Zhao, Lili Dong, Yongshi Wang, Dehong Kong, Chunqiang Hu, Yixiu Liang, Haiyan Chen, Wufeng Xue, Cuizhen Pan, Dong Ni, Xianhong Shu

**Affiliations:** National-Regional Key Technology Engineering Laboratory for Medical Ultrasound, Guangdong Key Laboratory of Biomedical Measurements and Ultrasound Imaging, School of Biomedical Engineering, Shenzhen University Medical School, Shenzhen University, 1066 Xueyuan Road, Shenzhen 518060, China; Medical Ultrasound Image Computing (MUSIC) Laboratory, Shenzhen University, 1066 Xueyuan Road, Shenzhen 518037, China; Marshall Laboratory of Biomedical Engineering, Shenzhen University, 1066 Xueyuan Road, Shenzhen 518037, China; Department of Echocardiography, Zhongshan Hospital, Fudan University, No. 1609 Xietu Road, Xuhui District, Shanghai 200030, China; Shanghai Institute of Medical Imaging, Fudan University, Shanghai, China; Department of Cardiology, Shanghai Institute of Cardiovascular Disease, Zhongshan Hospital, Fudan University, Shanghai 200030, China; Department of Echocardiography, Zhongshan Hospital, Fudan University, No. 1609 Xietu Road, Xuhui District, Shanghai 200030, China; Shanghai Institute of Medical Imaging, Fudan University, Shanghai, China; Department of Cardiology, Shanghai Institute of Cardiovascular Disease, Zhongshan Hospital, Fudan University, Shanghai 200030, China; Department of Ultrasound, Huadong Hospital, Fudan University, Shanghai 200040, China; Department of Echocardiography, Zhongshan Hospital, Fudan University, No. 1609 Xietu Road, Xuhui District, Shanghai 200030, China; Department of Echocardiography, Zhongshan Hospital, Fudan University, No. 1609 Xietu Road, Xuhui District, Shanghai 200030, China; Department of Echocardiography, Zhongshan Hospital, Fudan University, No. 1609 Xietu Road, Xuhui District, Shanghai 200030, China; Department of Echocardiography, Zhongshan Hospital, Fudan University, No. 1609 Xietu Road, Xuhui District, Shanghai 200030, China; Department of Echocardiography, Zhongshan Hospital, Fudan University, No. 1609 Xietu Road, Xuhui District, Shanghai 200030, China; Department of Cardiology, Shanghai Institute of Cardiovascular Disease, Zhongshan Hospital, Fudan University, Shanghai 200030, China; Department of Echocardiography, Zhongshan Hospital, Fudan University, No. 1609 Xietu Road, Xuhui District, Shanghai 200030, China; Shanghai Institute of Medical Imaging, Fudan University, Shanghai, China; Department of Cardiology, Shanghai Institute of Cardiovascular Disease, Zhongshan Hospital, Fudan University, Shanghai 200030, China; National-Regional Key Technology Engineering Laboratory for Medical Ultrasound, Guangdong Key Laboratory of Biomedical Measurements and Ultrasound Imaging, School of Biomedical Engineering, Shenzhen University Medical School, Shenzhen University, 1066 Xueyuan Road, Shenzhen 518060, China; Medical Ultrasound Image Computing (MUSIC) Laboratory, Shenzhen University, 1066 Xueyuan Road, Shenzhen 518037, China; Marshall Laboratory of Biomedical Engineering, Shenzhen University, 1066 Xueyuan Road, Shenzhen 518037, China; Department of Echocardiography, Zhongshan Hospital, Fudan University, No. 1609 Xietu Road, Xuhui District, Shanghai 200030, China; Shanghai Institute of Medical Imaging, Fudan University, Shanghai, China; Department of Cardiology, Shanghai Institute of Cardiovascular Disease, Zhongshan Hospital, Fudan University, Shanghai 200030, China; National-Regional Key Technology Engineering Laboratory for Medical Ultrasound, Guangdong Key Laboratory of Biomedical Measurements and Ultrasound Imaging, School of Biomedical Engineering, Shenzhen University Medical School, Shenzhen University, 1066 Xueyuan Road, Shenzhen 518060, China; Medical Ultrasound Image Computing (MUSIC) Laboratory, Shenzhen University, 1066 Xueyuan Road, Shenzhen 518037, China; Marshall Laboratory of Biomedical Engineering, Shenzhen University, 1066 Xueyuan Road, Shenzhen 518037, China; Department of Echocardiography, Zhongshan Hospital, Fudan University, No. 1609 Xietu Road, Xuhui District, Shanghai 200030, China; Shanghai Institute of Medical Imaging, Fudan University, Shanghai, China; Department of Cardiology, Shanghai Institute of Cardiovascular Disease, Zhongshan Hospital, Fudan University, Shanghai 200030, China

**Keywords:** mitral valve prolapse, Barlow’s disease, fbroelastic deficiency, deep learning, echocardiography, automated diagnosis

## Abstract

**Aims:**

To address the limitations of traditional diagnostic methods for mitral valve prolapse (MVP), specifically fibroelastic deficiency (FED) and Barlow’s disease (BD), by introducing an automated diagnostic approach utilizing multi-view echocardiographic sequences and deep learning.

**Methods and results:**

An echocardiographic data set, collected from Zhongshan Hospital, Fudan University, containing apical 2 chambers (A2C), apical 3 chambers (A3C), and apical 4 chambers (A4C) views, was employed to train the deep learning models. We separately trained view-specific and view-agnostic deep neural network models, which were denoted as MVP-VS and MVP view-agonistic (VA), for MVP diagnosis. Diagnostic accuracy, precision, sensitivity, F1-score, and specificity were evaluated for both BD and FED phenotypes. MVP-VS demonstrated an overall diagnostic accuracy of 0.94 for MVP. In the context of BD diagnosis, precision, sensitivity, F1-score, and specificity were 0.83, 1.00, 0.90, and 0.92, respectively. For FED diagnosis, the metrics were 1.00, 0.83, 0.91, and 1.00. MVP-VA exhibited an overall accuracy of 0.95, with BD-specific metrics of 0.85, 1.00, 0.92, and 0.94 and FED-specific metrics of 1.00, 0.83, 0.91, and 1.00. In particular, the MVP-VA model using mixed views for training demonstrated efficient diagnostic performance, eliminating the need for repeated development of MVP-VS models and improving the efficiency of the clinical pipeline by using arbitrary views in the deep learning model.

**Conclusion:**

This study pioneers the integration of artificial intelligence into MVP diagnosis and demonstrates the effectiveness of deep neural networks in overcoming the challenges of traditional diagnostic methods. The efficiency and accuracy of the proposed automated approach suggest its potential for clinical applications in the diagnosis of valvular heart disease.

## Graphical Abstract

**Graphical Abstract:**
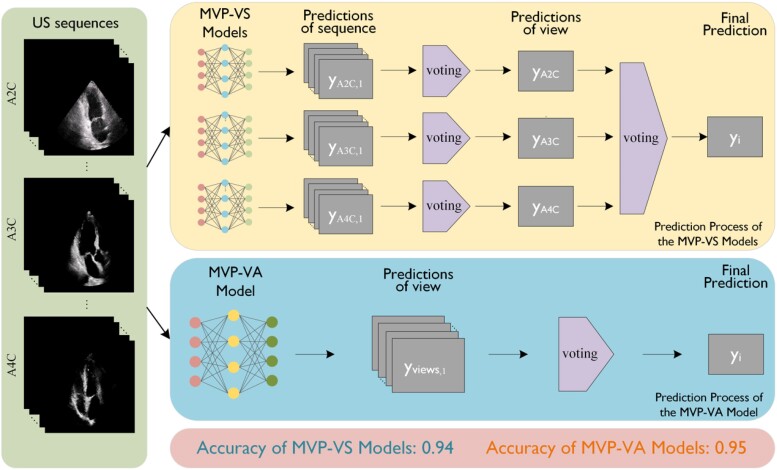

## Introduction

Mitral valve prolapse (MVP) affects 0.6–15% of the population and is associated with significant complications, including regurgitation and stroke.^1^ It is associated with various complications, including the progression of mitral regurgitation, endocarditis, sudden death, and stroke. MVP is defined as a systolic displacement (≥2 mm) of one or both mitral leaflets above the plane of the mitral annulus in the sagittal view of the mitral valve.^2,3^ The complexity of MVP is related to the complex anatomy of the mitral valve and the multiple factors that can contribute to its dysfunction. These factors may include leaflets abnormalities, elongated chordae tendineae, papillary muscles or annulus abnormalities, or a combination of them.^3,4^ Categorized into fibroelastic deficiency (FED) and Barlow’s disease (BD), the complexity of MVP makes an accurate diagnosis difficult.^5^ BD, a degenerative condition, involves myxomatous degeneration, leading to leaflet thickening with multi-segmental proposal, chordal elongation, and typical annular abnormalities. Typically asymptomatic, it manifests with a distinct murmur during exams.^4,5^ In contrast, FED, characterized by connective tissue deficiency, causes leaflets thinning and chords rupture with single-segment prolapse. Symptoms occur later in life, which distinguishes it from BD.^5,6^

Transthoracic echocardiography (TTE) is the first-line and most commonly used imaging modality for the diagnosis of MVP. In particular, detailed analysis of mitral valve lesions to define FED or BD usually requires three-dimensional (3D) transesophageal echocardiography (TEE).^7^ First, in cases of severe MR with surgical indication, this precise morphological evaluation of the mitral valve is necessary for surgical planning. This may prevent patients with non-severe MR from receiving comprehensive evaluation and ensure a precise diagnosis using 3D TEE in routine practice. Secondly, although 3D TEE and its derived volumetric measurements of valve anatomy can accurately differentiate these two aetiologies, it requires more expertise and is time-consuming.^8,9^ However, manual identification of scallop prolapse on 2D TTE is less effective, with an accuracy of 70.4%. In addition, MVP is significantly mis-diagnosed in those merely involving non-P2 scallop.^10,11^

To address this gap, our study pioneers a deep learning approach for automated MVP diagnosis using echocardiographic videos of multiple views. In recent years, researchers have made significant progress in leveraging echocardiography and machine learning (deep learning) for diagnosing heart valve diseases. Elalfi *et al*.^12^ proposed a pioneering method for diagnosing heart valve diseases using medical echocardiography images. Texture features are fed into an artificial neural network (ANN) classifier trained on a data set of 120 echocardiography images categorized into eight classes of heart valve diseases, achieving an impressive overall accuracy rate of 93.75%. Yang *et al*.^13^ conducted a study focusing on developing a three-step deep learning framework for automatic analysis of echocardiogram videos to screen for valvular heart disease (VHD). Vafaeezadeh *et al*.^14^ introduced an automated system for Carpentier’s classification using apical 4 chambers (A4C) and parasternal long-axis echocardiographic images and deep learning techniques. Deep learning techniques have also show promising capabilities in left ventricle quantification,^15,16^ ejection fraction estimation,^17–19^ mitral regurgitation quantification,^20^ etc. To the best of our knowledge, there is no existing work that explores the feasibility of deep learning and multi-view echocardiography for MVP diagnosis. Existing studies for automatic MVP diagnosis include electrocardiogram-based machine learning for MVP risk assessment. Tison *et al*.^21^ demonstrated robust predictive capabilities for complex ventricular ectopy using a deep convolutional neural network. Lin *et al*.^21,22^ extended this approach to young adults and showed promising results for early MVP prediction. Another study by Rajeshwari *et al*.^23^ investigated a phonocardiogram-based framework, achieving high accuracy and clinical interpretability.

In this research, leveraging the capabilities of deep learning, we aim to establish an automatic pipeline for a diagnosis of MVP from echocardiographic videos of multiple views. We present two models, namely MVP-VS and MVP view-agonistic (VA), leveraging different ultrasound views during model training. MVP-VS focuses on a single specific view, while MVP-VA combines various views during training to improve diagnostic efficiency.

## Methods

### Study population

Patients who underwent a TTE study at Zhongshan Hospital, Fudan University, between 1 January 2019 and 30 September 2020 were reviewed to identify the cases of MVP and healthy controls. This study was approved by the Ethics Review Committee of Zhongshan Hospital, Fudan University. Written informed consent was waived for the retrospective review of imaging obtained in the course of standard care. The inclusion criteria of patients with MVP were as follows: (i) presence of MVP with a diagnosis of FED or BD and (ii) at least mild mitral regurgitation. The exclusion criteria of patients with MVP were as follows: (i) presence of a prosthetic valve or history of mitral valve repair; (ii) concomitant mitral valve vegetations or perforation; (iii) mixed with functional mitral regurgitation; and (iv) compromised image quality that was not feasible for an accurate diagnosis. Healthy controls were defined as having normal heart size and function and no any VHD. The exclusion criteria for healthy controls, defined as normal group (NM), referred to items (i) and (iv) from the exclusion criteria of patients with MVP. A junior cardiologist reviewed and selected the studies according to the inclusion and exclusion criteria. The diagnosis of mitral valve disease (BD, FED, or NM) was further confirmed by an expert with over 15 years of experience in the echocardiographic diagnosis of mitral valve disease. The final data set comprised echocardiogram (echo) videos of 473 subjects, including 123 patients diagnosed with BD, 60 patients diagnosed with FED, and 308 healthy controls (normal). Each patient’s data included 1–3 standard 2D views: apical 2 chambers (A2C), apical 3 chambers (A3C), and A4C.

**Figure 1:**
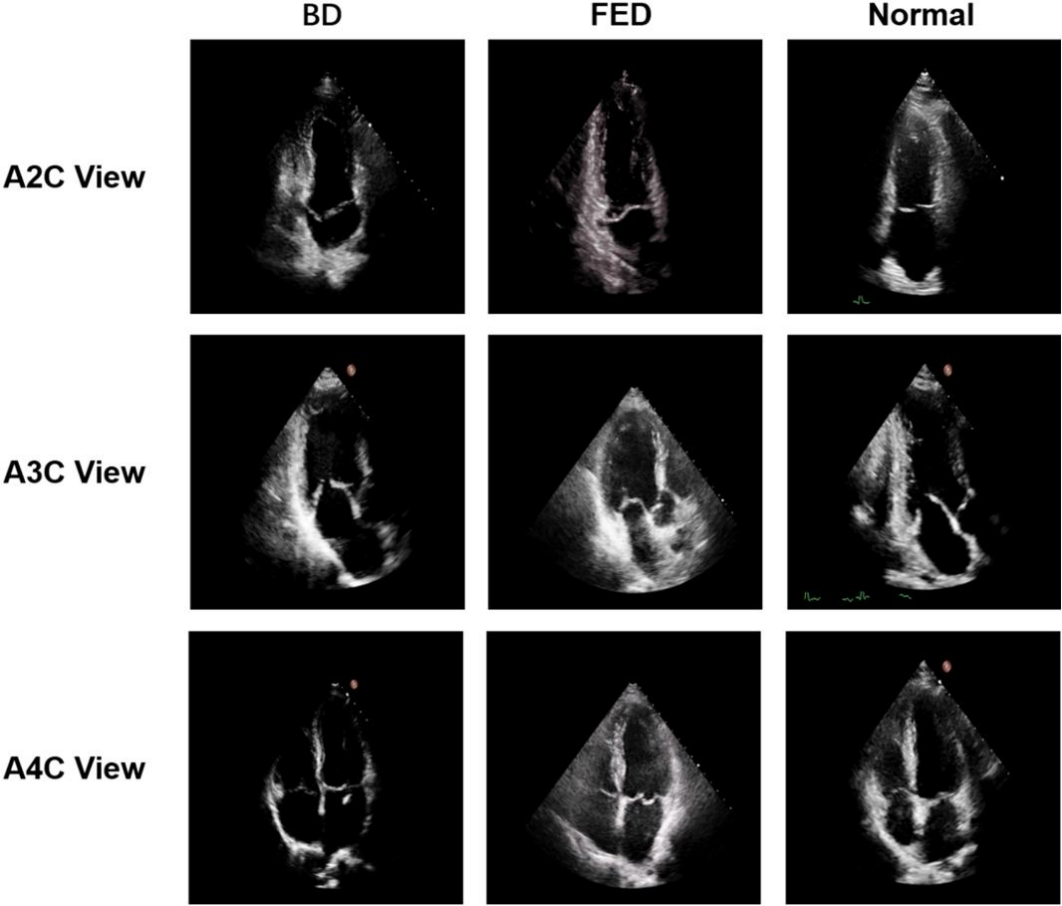
The image presents a sample echocardiographic image of the different types of MVP and healthy controls in A2C, A3C, and A4C views.

*Table 1* shows summary statistics for each disease type and views in the database. *Figure 1* presents a sample echocardiographic image of the different types of MVP and healthy controls in A2C, A3C, and A4C views.

**Table 1:** Number of videos for each disease type under various views in the database

	Disease type		
View	BD	FED	Normal
A2C	178	58	233
A3C	168	76	209
A4C	350	95	270

#### Pre-processing

All echo videos were deidentified before further processing. For the echocardiographic video of each view, pre-processing steps included temporal samples, intensity normalization, and resizing. For each video, eight frames were uniformly selected as the input of the network. The intensity was normalized to the range of [−1,1], and all images were resized to the size of 224 × 224 by cropping and interpolation.

#### Splitting

**Figure 2:**
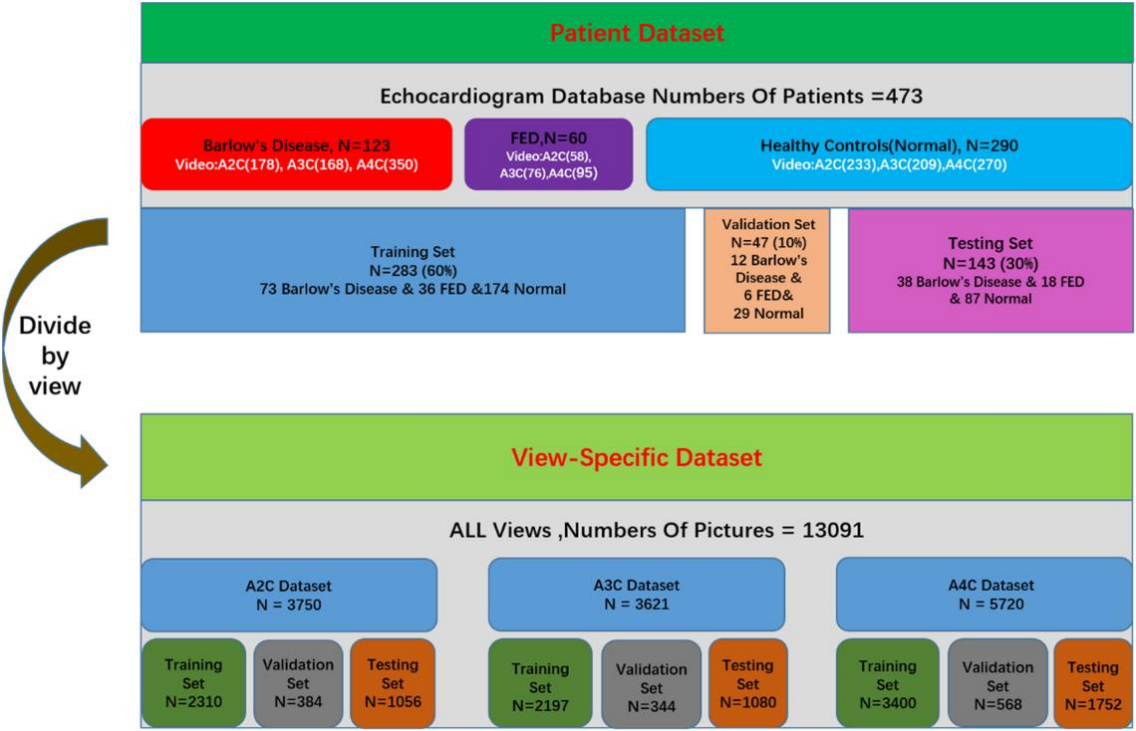
The sample quantities in the patient data set and view-specific data set.

The data set was randomly divided into training (60%), validation (10%), and testing (30%) sets in a subject-wise manner, ensuring that frames from each subject were exclusively allocated to one subset. The same class distribution of FED, BD, and normal categories was maintained across all subsets. In the case of view-specific (VS) models, echo frames corresponding to each view were appropriately allocated across the subsets (*Figure 2*).

### View-specific model

**Figure 3:**
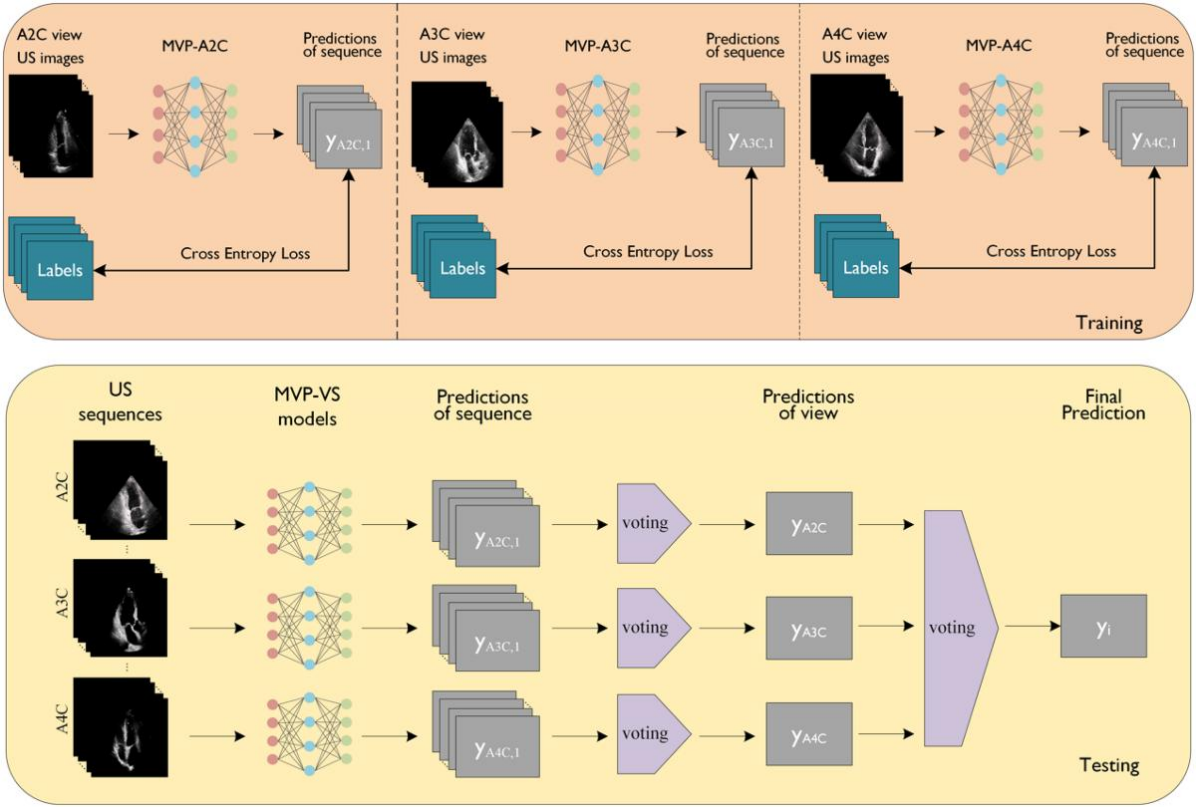
The training and testing process of MVP-VS model.

In this work, we employ the well-known ResNet18 model^24^ for the task of MVP diagnosis. To capture distinct features from different views, we initially trained separate ResNet18 models for each echocardiographic view. We denote them as MVP-VS models, which include MVP-A2C, MVP-A3C, and MVP-A4C. Once these view-specific models are trained, the voting results from multiple view-specific models are used as the final prediction. *Figure 3* illustrates the training and testing process of the MVP-VS model.

### View-agonistic model

**Figure 4:**
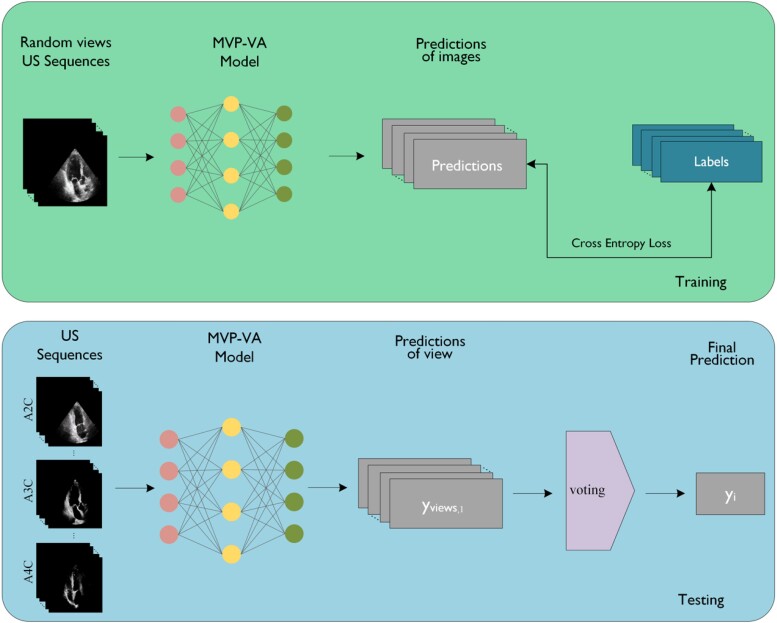
The training and testing process of MVP-VA model.

Building upon our previous approach, we introduced a novel method to comprehensively capture information across different views. In this new approach, we trained ResNet18 models using data from all views available for each patient regardless of which view it came from. We denote this new model as MVP-VA model. The MVP-VA model utilized the same training, validation, and test sets as the MVP-VS models. This approach aimed to achieve improved diagnosis performance with more training samples and capture the common and intrinsic features across all views. *Figure 4* illustrates the training and testing process of the MVP-VA model.

### Training details

We use ResNet18 as the backbone for both MVP-VS and MVP-VA models. ResNet18, a convolutional neural network with 18 layers, includes convolutional layers, batch normalization, ReLU activation, and residual blocks to mitigate the vanishing gradient problem.^24^ The categorical cross-entropy loss function was used as the optimization objectives in all model training. The Adam optimizer, with a learning rate of 10-4, a momentum of 0.9, and a weight decay of 10-4, was employed. Each network is trained for 50 epochs using a batch size of 32 and cross-entropy loss for supervision. All experiments were conducted on a NVIDIA GeForce RTX 3060 GPU.

### Evaluation metrics

We conducted performance evaluation for both view-specific models and view-agnostic models for patient-level MVP diagnosis. The patient-level diagnosis is obtained by majority voting of the predictions for all views from both VS and VA models. Evaluation metrics include accuracy, precision, sensitivity, F1-score, and specificity.

Accuracy: the ratio of correctly predicted samples to the total number of samples is:


(1) $$\begin{array}{c} \mathrm{Accuracy}=\frac{\mathrm{Number}\;\mathrm{of}\;\mathrm{correct}\;\mathrm{predictions}}{\mathrm{Total}\;\mathrm{number}\;\mathrm{of}\;\mathrm{predictions}}\; \end{array}$$

Precision: the ratio of true-positive predictions to the total number of positive predictions is:


(2) $$\begin{array}{c} \mathrm{Precision}=\frac{\mathrm{True}\;\mathrm{positives}}{\mathrm{True}\;\mathrm{positives}+\mathrm{false}\;\mathrm{positives}} \end{array}$$

Recall: the ratio of true-positive predictions to the total number of actual positives is:


(3) $$\begin{array}{c} \mathrm{Recall}=\frac{\mathrm{True}\;\mathrm{positives}}{\mathrm{True}\;\mathrm{positives}+\mathrm{false}\;\mathrm{negatives}}\; \end{array}$$

F1-score: the harmonic mean of precision and recall is:


(4) $$\begin{array}{c} F1-\mathrm{Score}=2\;\times \;\frac{\mathrm{Precision}\times \mathrm{recall}}{\mathrm{Precision}+\mathrm{recall}} \end{array}$$

Specificity: the ratio of true-negative predictions to the total number of actual negatives is:


(5) $$\begin{array}{c} \mathrm{Specificity}=\frac{\;\mathrm{True}\;\mathrm{negatives}}{\mathrm{True}\;\mathrm{negatives}+\mathrm{false}\;\mathrm{positives}} \end{array}$$

## Results

### Performance of view-specific models

The experiment results of the VS models on the test set are presented in *Table 2*. As can be drawn from the table, the VS models achieve great diagnosis accuracy for MVP (0.88, 0.90, and 0.90 for the three VS models alone). For BD diagnosis, VS model of the A4C views achieves the best precision (0.90) and recall (0.92). For FED diagnosis, the VS models get better performance, with the highest precision (0.95) and recall (1.0) achieved by A2C views. When the three VS models are combined, even higher performance can be achieved, as shown in the first block of *Table 2*. The total accuracy is 0.94, and the average precision and recall for BD, FED, and normal are clearly improved. This reveals the complementary information contained in each view for MVP diagnosis.

**Table 2:** Performance of MVP-VS models on the patient testing set

View	Class	Accuracy	Precision	Recall	F1-score	Specificity
Joint diagnosis	BD	0.94	0.83	1.00	0.90	0.92
	FED		1.00	0.83	0.91	1.00
	Normal		1.00	0.94	0.97	1.00
A2C	BD	0.88	0.84	0.82	0.83	0.92
	FED		0.95	1.00	0.97	0.99
	Normal		0.90	0.90	0.90	0.88
A3C	BD	0.90	0.84	0.91	0.87	0.90
	FED		0.93	0.92	0.92	0.98
	Normal		0.95	0.90	0.93	0.96
A4C	BD	0.90	0.90	0.92	0.91	0.89
	FED		0.98	0.90	0.94	0.96
	Normal		0.91	0.87	0.89	0.96

### Performance of view-agnostic model

*Table 3* illustrates the diagnosis performance of the MVP-VA model when applied to each view separately and to all views of the patient together on the test set. For multi-view diagnosis, our MVP-VA model demonstrated a commendable overall accuracy of 0.95. In particular, MVP-VA exhibited improved precision, sensitivity, and F1-score for both BD and FED diagnosis. For BD, the model performed well with a precision of 0.85, a recall of 1.00, and an F1-score of 0.92. For FED, the model achieved great performance with a precision of 1.0, a recall of 0.83, and an F1-score of 0.91. When the MVP-VA model is applied to each view separately, it can still achieve an overall accuracy above 0.90 for all the three views.

**Table 3:** Performance of MVP-VA model on the patient testing set

View	Class	Accuracy	Precision	Recall	F1-score	Specificity
All views joint diagnosis	BD	0.95	0.85	1.00	0.92	0.94
	FED		1.00	0.83	0.91	1.00
	Normal		1.00	0.96	0.98	1.00
A2C	BD	0.91	0.84	0.90	0.87	0.91
	FED		0.99	0.88	0.93	1.00
	Normal		0.94	0.92	0.93	0.93
A3C	BD	0.91	0.86	0.90	0.88	0.92
	FED		1.00	0.86	0.92	1.00
	Normal		0.92	0.94	0.93	0.93
A4C	BD	0.90	0.87	0.93	0.90	0.88
	FED		0.99	0.86	0.92	1.00
	Normal		0.91	0.88	0.89	0.94

## Discussion

In this study, we have pioneered an innovative automated approach for the diagnosis of MVP using deep convolutional neural networks and multi-view echocardiographic data. By introducing two models, namely MVP-VS and MVP-VA, our work aims to process multiple-view echocardiographic videos for MVP diagnosis. Both models achieve good performance for MVP diagnosis, with the view-agnostic model performing better.

Our primary objective was to improve diagnostic accuracy and reliability, thereby providing clinicians and patients with improved diagnostic and treatment options. The good performance of the MVP-VS model for each distinct view, with the lowest accuracy of 0.88, allows rapid and accurate MVP diagnosis even with only one echocardiographic video. For BD, the best precision and recall are achieved by the A4C view, while for FED, A4C achieves the best precision and A2C the best recall. This suggests that combing diagnostic results from multiple views can reduce false diagnosis and is consistent with the observations in the guideline^3^ that the four-chamber view alone is limited for diagnosis of MVP.

The MVP-VA model exhibits further enhanced diagnostic performance, surpassing the MVP-VS models. The MVP-VA model successfully captures the MVP-related information more comprehensively from the larger pool of all views, revealing that the view type information may play an ignorable role in the MVP diagnosis. The identification of MVP from any view can confirm the MVP diagnosis. In this case, there is no need to develop the view-specific model for each view separately. Combining videos from all views together and training a view-agnostic model can not only improve the diagnostic performance, but also be more computationally efficient and more flexible for clinical deployment.

Despite the high overall accuracy rates, we would like to examine the misclassified cases by our model. The MVP-VS model, which relies on specific echocardiographic views, tended to misclassify FED as BD. This occurred when the A3C view did not clearly display signs of prolapse, while the A4C view presented more typical FED features. These inconsistencies suggest that certain views may not always capture the full spectrum of disease characteristics, leading to model confusion. Similarly, the MVP-VA model, which integrates information from multiple views, showed a pattern of misclassification. In this model, FED was sometimes incorrectly identified as BD, likely due to overlapping features between the two conditions, especially in cases where leaflet length and prolapse extent were ambiguous. In addition, some normal cases were misclassified as BD, possibly due to poor image quality or the inherent complexity of multi-view data interpretation.

Despite the success of the proposed methods, it is essential to acknowledge the inherent limitations that shape the scope of our findings. The diversity of pathologies present, coupled with the limited data, may influence the model’s accuracy, especially when applied to real clinical scenarios. The complexity of clinical experience and patient-specific characteristics, pivotal in actual medical practice, remain a challenge to be fully encapsulated within our current model. Enhancing the robustness of our model requires collecting a wider spectrum of clinical data. Besides, our models are based on static images, which lack the temporal dynamic information of MV. By broadening the data set with diverse patient profiles, incorporating temporal dynamic information from echocardiographic videos, and refining the model’s algorithms, we can strive for a more comprehensive cardiac evaluation and gain clinically relevant insights into MVP and other cardiac conditions.

Looking forward, future research will focus on optimizing model performance and improving practicality. The exploration of additional deep learning architectures, feature extraction, and temporal modelling methods holds promise to further improve the model’s capabilities. Furthermore, the construction of a larger clinical data set with more diversity in terms of pathology will be our future focus, in order to validate and iterate the model’s capability and generalization, and translate it into real-world clinical applications.

## Conclusion

In this study, we have introduced an automated diagnostic approach leveraging deep learning for the precise classification of MVP. Utilizing multi-view echocardiographic data and the ResNet18 network, we developed two models, namely MVP-VS and MVP-VA, which aim to accurately distinguish between BD, FED, and normal cases. Our research not only delves into the significance of multi-view data in disease diagnosis but also highlights the substantial potential of deep learning in the realm of diagnosis of heart disease. The experimental results of both the MVP-VS and MVP-VA models affirm the promising outcomes in the task of diagnosing MVP. The MVP-VS models demonstrate the capability to accurately classify the disease with only a single view. Furthermore, in the scenario without views differentiation, the MVP-VA model demonstrated superior performance, indicating its ability to more effectively extract features related to MVP and to integrate multi-view features, and thereby enhancing the diagnostic accuracy. The high accuracy and F1-score achieved by the MVP-VA model on the patient test set provide strong evidence supporting the efficacy of our approach. In summary, our research introduces a pioneering automated diagnostic approach to the field of diagnosis of heart disease, providing a great potential for precise and personalized diagnosis in future medical diagnostics.

## Data Availability

The data underlying this article cannot be shared publicly due to ethical restrictions. Anonymized data are available to researchers on reasonable request to the corresponding author.

## Lead author biography

Dr Cuizhen Pan currently severs as a professor of echocardiography in Zhongshan Hospital, Fudan University. As a senior researcher, she devotes herself to investigate the cardiac structure and function in cardiovascular disease by advanced echocardiography techniques, aiming to further the mechanistic understanding of VHD, and improve the diagnose and management of patients with VHD.
